# Efficacy and safety of modified fluorouracil/leucovorin plus irinotecan and oxaliplatin (mFOLFIRINOX) compared with S‐1 as second‐line chemotherapy in metastatic pancreatic cancer

**DOI:** 10.1002/jgh3.12555

**Published:** 2021-05-10

**Authors:** Kenji Ikezawa, Ryosuke Kiyota, Ryoji Takada, Kazuma Daiku, Shingo Maeda, Toshihiro Imai, Yutaro Abe, Yugo Kai, Takuo Yamai, Nobuyasu Fukutake, Tasuku Nakabori, Reiko Ashida, Hiroyuki Uehara, Takahiro Tabuchi, Kazuhiro Katayama, Kazuyoshi Ohkawa

**Affiliations:** ^1^ Department of Hepatobiliary and Pancreatic Oncology Osaka International Cancer Institute Osaka Japan; ^2^ Department of Cancer Survey and Gastrointestinal Oncology Osaka International Cancer Institute Osaka Japan; ^3^ Cancer Control Center Osaka International Cancer Institute Osaka Japan

**Keywords:** adverse event, overall survival, pancreatic adenocarcinoma, peripheral sensory neuropathy, progression‐free survival

## Abstract

**Background and Aim:**

The optimal standard second‐line chemotherapy for metastatic pancreatic cancer (MPC) remains unclear. Here, we evaluated the efficacy and safety of modified fluorouracil/leucovorin plus irinotecan and oxaliplatin (mFOLFIRINOX) compared with oral fluoropyrimidine S‐1 as a second‐line chemotherapy in patients with MPC.

**Methods:**

We retrospectively reviewed 76 consecutive patients with metastatic pancreatic adenocarcinoma who underwent mFOLFIRINOX or S‐1 treatment as a second‐line chemotherapy after gemcitabine plus nab‐paclitaxel (GnP) failure at our department between December 2014 and February 2019.

**Results:**

Patients who underwent mFOLFIRINOX treatment exhibited significantly better objective response rates (ORRs) and progression‐free survival (PFS) than S‐1 (ORR, 20.0% *vs* 0%, *P* = 0.003; PFS, 3.7 *vs* 2.1 months, *P* = 0.010). Although baseline patient characteristics of age, performance status, and serum albumin levels differed significantly between the two groups, mFOLFIRINOX was identified as an independent factor of favorable PFS on multivariate analyses. Grade 3–4 neutropenia and peripheral sensory neuropathy occurred more frequently in the mFOLFIRINOX group. The median overall survival from the initiation of second‐line chemotherapy was not significantly longer in the mFOLFIRINOX group than in the S1 group (8.5 *vs* 5.8 months, respectively; *P* = 0.213); however, the 8‐month survival rate was significantly higher in the mFOLFIRINOX group (56.0% *vs* 27.5%, respectively; *P* = 0.030).

**Conclusions:**

mFOLFIRINOX as a second‐line regimen contributed to favorable treatment outcomes, but induced more frequent adverse events than S‐1. On multivariate analyses, mFOLFIRINOX was identified as an independent factor with favorable PFS, suggesting that mFOLFIRINOX could be a promising treatment option for patients with GnP failure.

## Graphical abstract

The optimal standard second‐line chemotherapy for metastatic pancreatic cancer (MPC) remains unclear. In this retrospective study for MPC patients with gemcitabine plus nab‐paclitaxel (GnP) failure, modified fluorouracil/leucovorin plus irinotecan and oxaliplatin (mFOLFIRINOX) as a second‐line regimen contributed to favorable treatment outcomes but induced more frequent adverse events than S‐1. On multivariate analyses, mFOLFIRINOX was identified as an independent factor with favorable progression‐free survival, suggesting that mFOLFIRINOX could be a promising treatment option for patients with GnP failure.

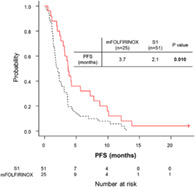

## Introduction

Pancreatic cancer (PC) has become the third leading cause of cancer‐related death in the United States, and the incidence of PC continues to increase.^1^, ^2^ PC has a dismal prognosis, with a 5‐year overall survival rate of 9%,^1^ and early detection of PC remains difficult.^3^, ^4^ Approximately 80% of patients with PC have metastatic or locally advanced PC, and these patients primarily undergo chemotherapy.^5^ As a first‐line chemotherapy, combination chemotherapy has become standard in advanced PC after the superiority of gemcitabine (GEM) plus nab‐paclitaxel (GnP), and fluorouracil/leucovorin plus irinotecan and oxaliplatin (FOLFIRINOX) over GEM monotherapy was demonstrated in multicenter phase III studies.^6^, ^7^ Although standard FOLFIRINOX has greater effectiveness, management of adverse events is difficult because of high rates of grade 3/4 neutropenia and febrile neutropenia.^7^, ^8^ A modified FOLFIRINOX regimen (mFOLFIRINOX; no or decreased administration of bolus 5‐fluorouracil [5‐FU] plus decreased irinotecan administration) has been shown to exhibit improved safety with maintained efficacy.^9^, ^10^ Although there have been no direct, prospective, randomized comparisons between the original FOLFIRINOX regimen and mFOLFIRINOX, this modified regimen is thought to be equivalent to the original FOLFIRINOX regimen for the palliative treatment of PC.^10^, ^11^, ^12^

Second‐line chemotherapy can improve the survival of patients with metastatic pancreatic cancer (MPC) after failure of first‐line GEM‐based chemotherapy.^13^, ^14^, ^15^, ^16^ Although several previous phase III studies demonstrated survival benefit with their study regimens,^14^, ^15^, ^16^ the standard treatment remains to be established. Although mFOLFIRINOX following GnP failure could be a promising strategy because both GnP and mFOLFIRINOX have shown favorable outcomes, limited data are available regarding sequential therapy with GnP and mFOLFIRINOX.^17^ In Japan, S‐1, an oral fluoropyrimidine, has been widely used as a second‐line therapy because S‐1 has antitumor activity with tolerable toxicity against GEM‐refractory PC.^18^, ^19^, ^20^, ^21^

In this study, we aimed to examine the efficacy and safety of mFOLFIRINOX in comparison with S‐1 as a second‐line chemotherapy in patients with metastatic adenocarcinoma of the pancreas after GnP failure.

## Methods

### Study design and patients

We retrospectively reviewed the clinical data for 76 consecutive patients with pathologically proven metastatic adenocarcinoma of the pancreas who underwent chemotherapy using mFOLFIRINOX or S‐1 as a second‐line regimen after GnP failure at our department between December 2014 and February 2019. mFOLFIRINOX was given every 2 weeks as follows: 2 h intravenous infusion of oxaliplatin at 85 mg/m^2^ and 2 h intravenous infusion of l‐leucovorin at 200 mg/m^2^, intravenous infusion of irinotecan over 90 min at 150 mg/m^2^, followed by a continuous intravenous infusion of 5‐FU over 46 h at 2400 mg/m^2^, with an omission of bolus 5‐FU infusion. S‐1 was administered orally twice a day at a dose of 40 mg/m^2^ for 4 weeks in a 6‐week cycle. Second‐line regimens were decided based on the general conditions of the patients and willingness to undergo aggressive therapy. The dosages and schedules of chemotherapeutic drugs were adjusted at the discretion of each physician according to the conditions of the patients. For each patient, data were collected regarding age, sex, Eastern Cooperative Oncology Group (ECOG) performance status (PS), primary tumor location, metastatic site number, lymph node involvement, biliary drainage, neutrophil‐to‐lymphocyte ratio (NLR), carbohydrate antigen 19–9 (CA19‐9) levels, carcinoembryonic antigen (CEA) levels, UDP glucuronosyltransferase family 1 member A1 (UGT1A1) status, treatment details (chemotherapeutic regimens, treatment response, and toxicities), and survival time. Tumor responses were graded according to the Response Evaluation Criteria in Solid Tumor (RECIST) ver. 1.1.^22^ Hematological and nonhematological adverse events were graded according to the Common Terminology Criteria of Adverse Events version 4.0. Progression‐free survival (PFS) was calculated from the administration date for the first dose of chemotherapy to the date of disease progression or any cause of death, whichever occurred first. Overall survival (OS) was calculated from the initiation of second‐line chemotherapy to the date of death due to any cause. We also examined 8‐month survival rates, which were set according to the median OS in previous reports on single‐arm FOLFIRINOX as a second‐line chemotherapy.^17^, ^23^, ^24^ Data from patients who were alive at the end of the follow‐up period (December 2020) were censored. The study protocol was approved by the Institutional Review Board at Osaka International Cancer Institute (approval no. 18225‐4), and the study was performed in accordance with the Declaration of Helsinki.

### Statistical analysis

Categorical variables are described as percentages, and continuous variables are presented as the median and range. Patient characteristics, treatment outcomes, toxicities of second‐line chemotherapy, and the proportion of the patients who underwent third‐line chemotherapeutic regimens were compared using Chi‐square and Fisher's exact tests for categorical variables or Mann–Whitney *U*‐test for continuous variables. Analyses of OS and PFS were performed using the Kaplan–Meier method, and differences were evaluated using log‐rank tests.

Using the Cox proportional hazard model, univariate and multivariate analyses were performed to identify significant prognostic factors associated with PFS. The following 14 variables were examined: age, sex, ECOG PS, body mass index, primary tumor location, metastatic site number, lymph node involvement, biliary drainage, NLR, CA19‐9, CEA, albumin, creatinine, and second‐line chemotherapy regimens.

Hazard ratios (HRs) and 95% confidence intervals (CIs) were calculated. Factors with *P* values less than 0.20 in univariate analysis were entered into multivariate Cox models. Statistical analyses were performed using EZR (Saitama Medical Center, Jichi Medical University, Saitama, Japan), a graphical interface for the R Commander software package for Windows (version 1.50).^25^ Results with *P* values less than 0.05 were considered significant.

## Results

### Patient characteristics

The characteristics of the 76 patients included in the current study are summarized in Table 1. The median age was 65.5 years (range, 43–81 years), and 35 patients (46.1%) were men. ECOG PS was 0 in 30 patients (39.5%), 1 in 38 patients (50.0%), and 2 in eight patients (10.5%). Median body mass index was 21.3 kg/m^2^ (range, 15.3–28.8 kg/m^2^). The primary tumor sites were the pancreas head in 39 patients (51.3%) and the pancreas body/tail in 37 patients (48.7%). The number of metastatic sites was 1–3 in 20 patients (26.3%) and 4 or more in 56 patients (73.7%). Lymph node involvement was diagnosed in 23 patients (30.3%), and biliary drainage was performed in 30 patients (39.5%). NLR was less than 3 in 27 patients (35.5%). The median levels of CA19‐9, CEA, albumin, and creatinine were 1051 IU/mL (range, 2–100 000 IU/mL), 6.85 ng/mL (range, 1.5–1743 ng/mL), 3.55 mg/dL (range, 2.3–4.6 mg/dL), and 0.70 mg/dL (range, 0.33–1.33 mg/dL), respectively.

**Table 1:** Baseline characteristics of the study patients

	Total	S1	mFOLFIRINOX	*P* value
	(*n* = 76)	(*n* = 51)	(*n* = 25)	
Age, median (range), years	65.5 (43–81)	69 (47–81)	60 (43–70)	**<0.001** ^†^
Sex				
Male, *n* (%)	35 (46.1)	22 (43.1)	13 (52.0)	0.629^‡^
Female, *n* (%)	41 (53.9)	29 (56.9)	12 (48.0)	
ECOG PS				
0, *n* (%)	30 (39.5)	15 (29.4)	15 (60.0)	**0.021** ^‡^ (PS 0 vs. PS 1–2)
1, *n* (%)	38 (50.0)	28 (54.9)	10 (40.0)	
2, *n* (%)	8 (10.5)	8 (15.7)	0 (0)	
Body mass index, median (range), kg/m^2^	21.3 (15.3–28.8)	21.0 (15.3–28.8)	21.5 (16.3–27.1)	0.359^†^
Primary tumor location				
Head, *n* (%)	39 (51.3)	24 (47.1)	13 (52.0)	0.872^‡^
Body/tail, *n* (%)	37 (48.7)	27 (52.9)	12 (48.0)	
The number of metastatic sites				
1–3	20 (26.3)	12 (23.5)	8 (32.0)	0.610^‡^
≧4	56 (73.7)	39 (76.5)	17 (68.0)	
Lymph node involvement				
No, *n* (%)	53 (69.7)	37 (72.5)	16 (64.0)	0.620^‡^
Yes, *n* (%)	23 (30.3)	14 (27.5)	9 (36.0)	
Biliary drainage				
No, *n* (%)	46 (60.5)	31 (60.8)	15 (60.0)	1^‡^
Yes, *n* (%)	30 (39.5)	20 (39.2)	10 (40.0)	
NLR				
<3	27 (35.5)	17 (33.3)	10 (40.0)	0.752^‡^
≧3	49 (64.5)	34 (66.7)	15 (60.0)	
CA19‐9, median (range), IU/mL	1051 (2–100 000)	1090 (2–100 000)	955 (2–24 734)	0.799^†^
CEA, median (range), ng/mL	6.85 (1.5–1743)	7.5 (1.7–1743)	4.9 (1.5–84.2)	0.138^†^
Albumin, median (range), mg/dL	3.55 (2.3–4.6)	3.4 (2.3–3.8)	3.7 (2.9–4.6)	**0.006** ^†^
Creatinine, median (range), mg/dL	0.70 (0.33–1.33)	0.71 (0.33–1.33)	0.63 (0.35–1.08)	0.207^†^
UGT1A1*28/UGT1A1*6				
Wild‐type	32 (42.1)	20 (39.2)	12 (48.0)	0.409^‡^ (wild vs. hetero/homo)
Heterozygote	21 (27.6)	10 (19.6)	11 (44.0)	
Homozygote	4 (5.3)	2 (3.9)	2 (8.0)	
Not measured	19 (25.0)	19 (37.3)	0 (0)	

^†^ Mann–Whitney *U*‐test.

^‡^ Chi‐square test.

Bold values indicate *P* < 0.05.

CA19‐9, carbohydrate antigen 19–9; CEA, carcinoembryonic antigen; ECOG, Eastern Cooperative Oncology Group; mFOLFIRINOX, modified fluorouracil/leucovorin plus irinotecan and oxaliplatin; NLR, neutrophil‐to‐lymphocyte ratio; PS, performance status.

Table 1 also shows a comparison of patient characteristics between the mFOLFIRINOX and S1 groups. We found no significant differences in sex, body mass index, primary tumor location, metastatic site number, lymph node involvement, histological type, NLR, CA19‐9, CEA, and UGT1A1 status (Table 1). By contrast, patients who underwent FOLFIRINOX were significantly younger (*P* < 0.001) and had significantly better ECOG PS (*P* = 0.021). Moreover, serum albumin levels were significantly higher in the mFOLFIRINOX group (*P* = 0.006).

### Treatment outcomes

**Figure 1:**
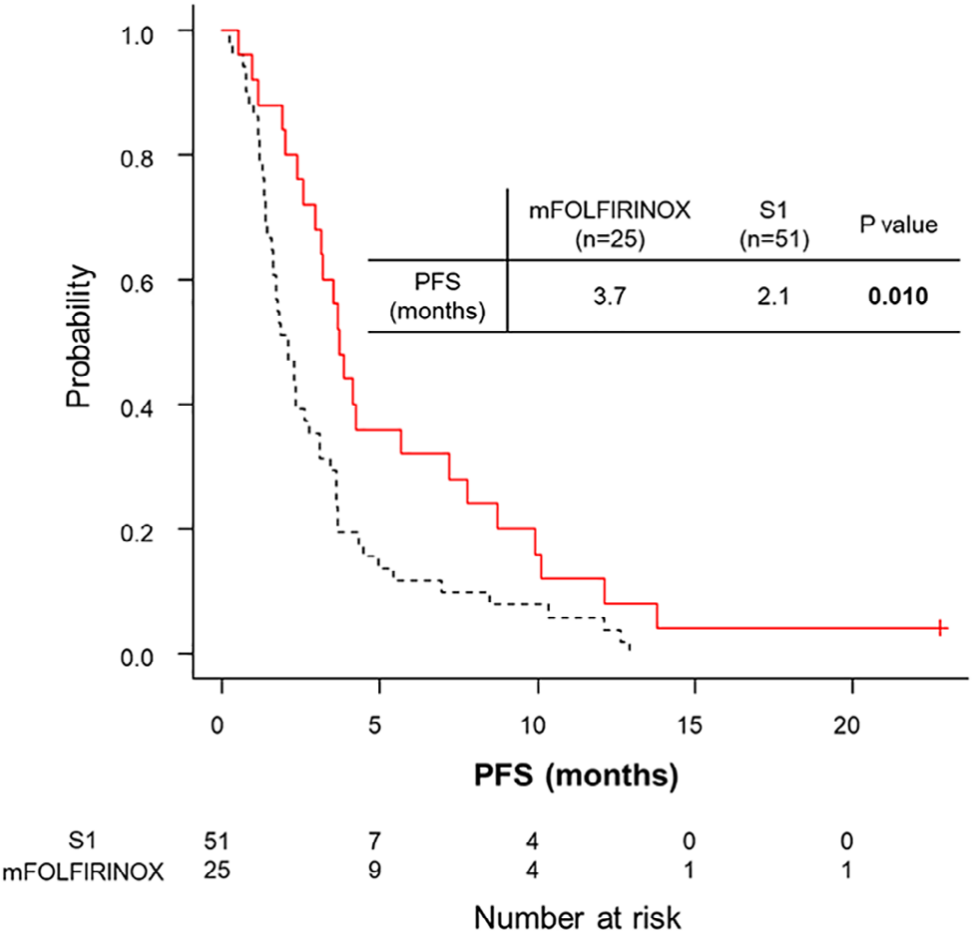
Comparison of progression‐free survival (PFS) after the initiation of second‐line chemotherapy. 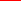, modified fluorouracil/leucovorin plus irinotecan and oxaliplatin (mFOLFIRINOX); 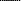, S1.

The overall response rate (ORR) of second‐line chemotherapy was 6.6% (5/76; complete response, 0; partial response, 6). ORR was significantly higher in the mFOLFIRINOX group than in the S1 group (20.0% [5/25] *vs* 0% [0/51]; *P* = 0.003). The disease control rate was not significantly different between the two groups (mFOLFIRINOX, 64.0% [16/25] *vs* S1, 51.0% [26/51]; *P* = 0.408). The median PFS of second‐line chemotherapy was 2.6 months (95% CI, 1.9–3.6 months). The median PFS was significantly longer in the mFOLFIRINOX group than in the S1 group (3.7 months [95% CI, 3.0–7.2 months] *vs* 2.1 months [95% CI, 1.6–2.8 months], *P* = 0.010; Fig. 1). Among the 68 study patients whose PS was 0–1, the median PFS was also significantly longer in the mFOLFIRINOX group than in the S1 group (3.7 months [95% CI, 3.0–7.2 months] *vs* 2.1 months [95% CI, 1.6–2.8 months], *P* = 0.010).

Overall, 26 patients (34.2%) received third‐line chemotherapy. The rate of patients who underwent third‐line chemotherapy was significantly higher in the mFOLFIRINOX group than in the S‐1 group (52.0% [13/25] *vs* 25.5% [13/51], respectively; *P* = 0.042; Table 2). The regimens of third‐line therapy in the mFOLFIRINOX group were GEM plus S‐1 in five patients, S‐1 in three patients, FOLFOX in two patients, GEM in one patient, GEM plus erlotinib in one patient, and S‐1 plus radiation for palliation of pain in one patient, whereas those in the S‐1 group were mFOLFIRINOX in nine patients, GEM plus S‐1 in one patient, FOLFOX in two patients, GEM in one patient, and GEM plus erlotinib in one patient.

**Table 2:** Treatment and outcomes of the study patients

	Total (*n* = 76)	S1 (*n* = 51)	mFOLFIRINOX (*n* = 25)	*P* value
Overall response rate, *n* (%)	5 (6.6)	0 (0)	5 (20.0)	0.003^†^
Disease control rate, *n* (%)	42 (55.3)	26 (51.0)	16 (64.0)	0.408^‡^
Third‐line chemotherapy, *n* (%)	26 (34.2)	13 (25.5)	13 (52.0)	0.042^‡^
Third‐line chemotherapy regimen				
mFOLFIRINOX, *n* (%)	9 (11.8)	9 (17.6)	—	
S‐1, *n* (%)	3 (3.9)	—	3 (12.0)	
Gemcitabine plus S‐1, *n* (%)	6 (7.9)	1 (2.0)	5 (20.0)	
FOLFOX, *n* (%)	3 (3.9)	1 (2.0)	2 (8.0)	
Gemcitabine, *n* (%)	2 (2.6)	1 (2.0)	1 (4.0)	
Gemcitabine plus erlotinib, *n* (%)	2 (2.6)	1 (2.0)	1 (4.0)	
S‐1 plus radiation, *n* (%)	1 (1.3)	0 (0)	1 (4.0)	

^†^ Fisher's exact test.

^‡^ Chi‐square test. Statistically significant at *P* < 0.05.

mFOLFIRINOX, modified fluorouracil/leucovorin plus irinotecan and oxaliplatin.

**Figure 2:**
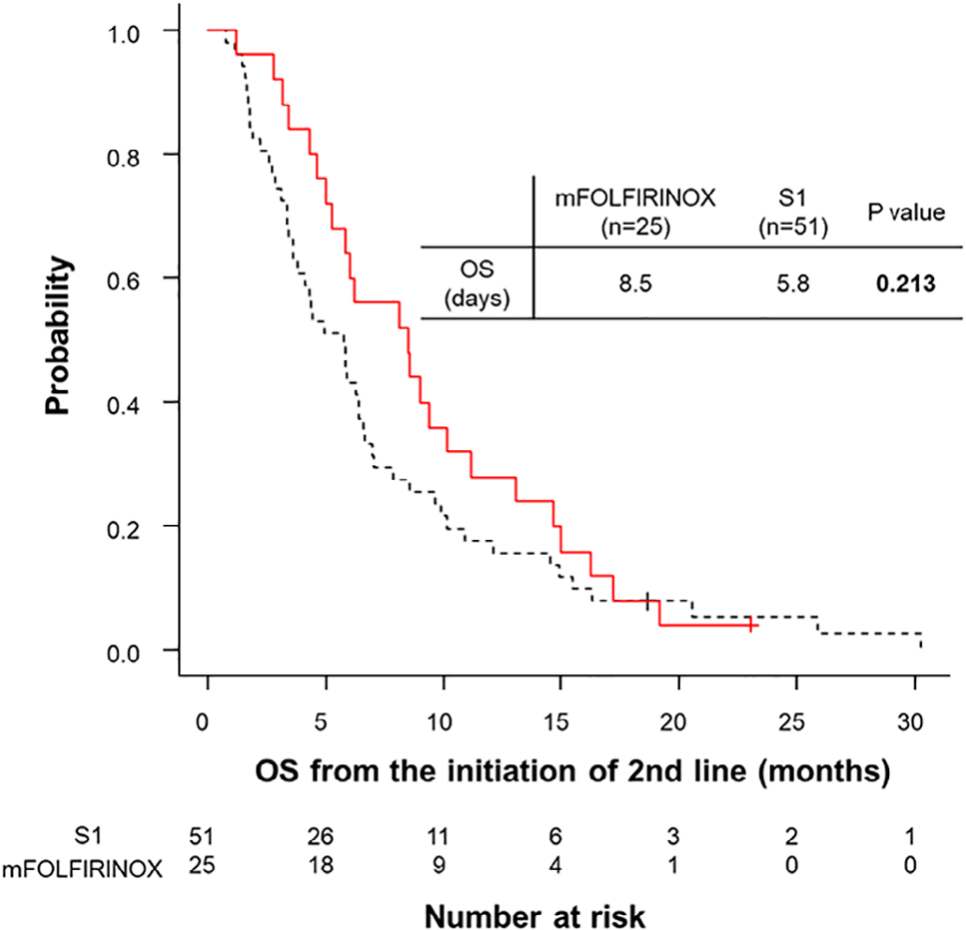
Comparison of overall survival (OS) from the initiation of second‐line chemotherapy. 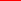, modified fluorouracil/leucovorin plus irinotecan and oxaliplatin (mFOLFIRINOX); 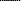, S1.

The median OS from the initiation of second‐line chemotherapy was 6.0 months (95% CI, 4.4–7.0 months). Among all patients, 74 patients (97.4%) had died at the end of the follow‐up period. The median OS was not significantly longer in the mFOLFIRINOX group than in the S1 group (OS, 8.5 months [95% CI, 5.3–11.1 months] *vs* 5.8 months [95% CI, 3.6–6.6 months], *P* = 0.213; Fig. 2). The 8‐month survival rate from the initiation of second‐line chemotherapy was significantly higher in the mFOLFIRINOX group than in the S1 group (56.0% [14/25] *vs* 27.5% [14/51], respectively; *P* = 0.030). Among the 68 study patients whose PS was 0–1, the median OS was comparatively longer in the mFOLFIRINOX group than in the S1 group (OS, 8.5 months [95% CI, 5.3–11.1 months] *vs* 5.8 months [95% CI, 3.8–7.0 months], *P* = 0.278).

### Safety

No treatment‐related deaths occurred. The toxicity profile is summarized in Table 3. The incidence rate of neutropenia was significantly higher in the mFOLRIFINOX group than in the S1 group (all grade, 88.0% *vs* 3.3%, respectively [*P* < 0.001]; grade 3 or 4, 52.0% *vs* 3.9%, respectively [*P* < 0.001]). Febrile neutropenia occurred in two cases (8.0%) in the mFOLFIRINOX group. The rate of patients who underwent granulocyte colony‐stimulating factor (G‐CSF) treatment was significantly higher in the mFOLFIRINOX group than in the S‐1 group (28.0% [7/25] *vs* 0% [0/51], respectively; *P* < 0.001). Among the major grade 3–4 nonhematological toxicities, the rate of peripheral sensory neuropathy (PN) was significantly higher in the mFOLFIRINOX group than in the S‐1 group (16.0% [4/25] *vs* 2.0% [1/51], respectively; *P* = 0.038). Among five patients who suffered from grade 3–4 PN, one patient had experienced grade 2 PN, and the other four patients had experienced grade 3 PN during GnP treatment.

**Table 3:** Toxicities in patients with unresectable pancreatic cancer treated with second‐line chemotherapy

	S1 (*n* = 51)	mFOLFIRINOX (*n* = 25)	*P* value
Anemia			
All grade (%)	49 (96.1)	24 (96.0)	1^†^
Grade 3, 4 (%)	9 (17.6)	6 (24.0)	0.729^‡^
Neutropenia			
All grade (%)	17 (33.3)	22 (88.0)	**<0.001** ^†^
Grade 3, 4 (%)	2 (3.9)	13 (52.0)	**<0.001** ^†^
Thrombocytopenia			
All grade (%)	19 (37.3)	14 (56.0)	0.193^‡^
Grade 3, 4 (%)	0 (0)	1 (4.0)	0.329^†^
FN	0 (0)	2 (8.0)	0.105^†^
Diarrhea			
All grade (%)	10 (19.6)	12 (48.0)	**0.022** ^‡^
Grade 3, 4 (%)	0 (0)	1 (4.0)	0.329^†^
Constipation			
All grade (%)	10 (19.6)	11 (44.0)	**0.050** ^‡^
Grade 3, 4 (%)	0 (0)	0 (0)	—
Decreased appetite			
All grade (%)	22 (43.1)	19 (76.0)	**0.014** ^‡^
Grade 3, 4 (%)	0 (0)	1 (4.0)	0.329^†^
Nausea			
All grade (%)	13 (25.5)	13 (52.0)	**0.042** ^‡^
Grade 3, 4 (%)	1 (2.0)	0 (4.0)	1^†^
Fatigue			
All grade (%)	16 (31.4)	17 (68.0)	**0.005** ^‡^
Grade 3, 4 (%)	0 (0)	0 (0)	—
PN			
All grade (%)	27 (52.9)	23 (92.0)	**<0.001** ^†^
Grade 3, 4 (%)	1 (2.0)	4 (16.0)	**0.038** ^†^

^†^ Fisher's exact test.

^‡^ Chi‐square test. Bold values indicate *P* < 0.05.

FN, febrile neutropenia; mFOLFIRINOX, modified fluorouracil/leucovorin plus irinotecan and oxaliplatin; PN, peripheral sensory neuropathy.

### Factors associated with PFS

Finally, we examined the predictive factors associated with PFS in patients receiving second‐line chemotherapy. In univariate analysis, two variables were found to be significantly associated with PFS, that is, serum albumin levels (HR, 0.550; 95% CI, 0.343–0.882; *P* = 0.013) and second‐line chemotherapy (HR, 0.526; 95% CI, 0.320–0.866; *P* = 0.012; Table 4). Multivariate analysis was performed using three variables (tumor location, serum albumin levels, and second‐line chemotherapy). Second‐line chemotherapy was identified as a statistically significant independent prognostic predictor (HR, 0.557; 95% CI, 0.336–0.925; *P* = 0.024; Table 4). Further in the multivariate analysis using the data of the 68 study patients whose PS was 0–1, second‐line chemotherapy was demonstrated as a statistically significant independent prognostic predictor (HR, 0.554; 95% CI, 0.329–0.933; *P* = 0.026).

**Table 4:** Univariate and multivariate analyses of factors associated with progression‐free survival from the initiation of second‐line chemotherapy

Factor	Univariate		Multivariate	
	HR (95% CI)	*P* value	HR (95% CI)	*P* value
Age				
<65 years	1			
≥65 years	1.330 (0.829–2.133)	0.237		
Sex				
Female	1			
Male	1.045 (0.663–1.647)	0.851		
ECOG PS				
0	1			
1 or 2	1.029 (0.645–1.641)	0.904		
Body mass index				
<20 kg/m^2^	1			
≥20 kg/m^2^	0.843 (0.516–1.375)	0.493		
Tumor location				
Body/tail	1			
Head	1.432 (0.897–2.287)	0.133	1.357 (0.846–2.176)	0.205
Number of metastatic sites				
1–3	1			
≧4	1.157 (0.612–1.938)	0.578		
Lymph node involvement				
No	1			
Yes	1.079 (0.654–1.781)	0.765		
Biliary drainage				
No	1			
Yes	1.152 (0.720–1.843)	0.556		
NLR				
<3	1			
≧3	1.118 (0.694–1.801)	0.647		
CA19‐9				
<1000 IU/mL	1			
≥1000 IU/mL	1.225 (0.778–1.929)	0.380		
CEA				
<10 ng/mL	1			
≥10 ng/mL	1.329 (0.816–2.165)	0.253		
Albumin				
<3.5 mg/mL	1			
≥3.5 mg /mL	0.550 (0.343–0.882)	**0.013**	0.631 (0.389–1.023)	0.062
Creatinine				
<0.7 mg/dL	1			
≥0.7 mg/dL	0.915 (0.581–1.440)	0.700		
Second‐line chemotherapy				
S1	1		1	
mFOLFIRINOX	0.526 (0.320–0.866)	**0.012**	0.557 (0.336–0.925)	**0.024**

Bold values indicate *P* < 0.05.

ALP, alkaline phosphatase; CEA, carcinoembryonic antigen; CI, confidence interval; ECOG, Eastern Cooperative Oncology Group; FBS, fasting blood sugar; HR, hazard ratio; MPD, main pancreatic duct; NLR, neutrophil‐to‐lymphocyte ratio; P‐amylase, pancreatic amylase; PS, performance status.

## Discussion

In previous studies in the single‐arm setting, the FOLFIRINOX regimen, including mFOLFIRINOX, showed favorable treatment outcomes as a second‐line chemotherapy after the failure of GEM‐based chemotherapy.^17^, ^23^, ^24^, ^26^, ^27^ Response rates, PFS, and OS in patients with MPC were 10.6–22.2%, 2.8–5.4 months, and 7.0–9.8 months, respectively.^17^, ^23^, ^27^ However, limited data exist regarding the direct comparison between FOLFIRINOX and fluoropyrimidine, including S‐1.^28^ In our current study of 78 patients with metastatic pancreatic adenocarcinoma after GnP failure, we observed significantly favorable response rates and PFS in the mFOLFIRINOX group compared with the S1 group. Since second‐line regimens were decided based on the general conditions of the patients and willingness to undergo aggressive therapy, several baseline characteristics were significantly different between the mFOLFIRINOX and S1 groups. To adjust for these differences, we performed multivariate analyses, demonstrating that mFOLFIRINOX was an independent prognostic factor with favorable PFS. Moreover, in the multivariate analysis using the data of the 68 study patients whose PS was 0–1, second‐line chemotherapy was identified as an independent prognostic predictor. Collectively, mFOLFIRINOX as a second‐line chemotherapy could contribute to prolonging PFS with favorable response rates.

In this study, we observed favorable OS from the initiation of second‐line therapy in the mFOLFIRINOX group compared with that in the S‐1 group, but did not find significant differences primarily because a few patients in the S‐1 group survived for a long time (more than 2 years). By contrast, the 8‐month survival rate was significantly higher in the mFOLFIRINOX group than in the S‐1 group, suggesting that mFOLFIRINOX may have the potential to demonstrate significant survival benefit as a second‐line chemotherapy in a larger‐scale setting. Nanoliposomal irinotecan (nal‐IRI) plus 5‐FU/l‐leucovorin (LV) was not used in the current study because it was approved in Japan during the study period. Because nal‐IRI plus 5‐FU/LV showed superiority over 5‐FU/LV as a second‐line chemotherapy for patients with MPC,^14^ this combination regimen could be a standard second‐line chemotherapy following GnP treatment failure. Recently, platinum‐containing regimens, including FOLFIRINOX, have been increasingly used owing to their contributions to a favorable OS in patients with homologous recombination deficiency, comprising approximately 20% of patients with PC.^29^ A direct comparison between mFOLFIRINOX and nal‐IRI plus 5‐FU/LV as a second‐line chemotherapy is necessary.

Notably, mFOLFIRINOX induced a higher rate of adverse events compared with S‐1. In the mFOLFIRINOX group, grade 3–4 neutropenia was more frequently observed. G‐CSF treatment was administered in 28.0% of patients in the mFOLFIRINOX group. Recent studies have demonstrated the efficacy of G‐CSF not only to reduce the risk of neutropenia but also to improve the survival of patients who underwent FOLFIRINOX treatment.^30^, ^31^ Appropriate use of G‐CSF could contribute to safe continuation of mFOLFIRINOX without severe infectious diseases. Among the nonhematological toxicities, the rate of grade 3–4 PN was significantly higher in the mFOLFIRINOX group (16.0%). In a single‐arm retrospective study of mFOLFIRINOX as a second‐line chemotherapy after GnP failure, the frequency of grade 3–4 PN was reported to be 10.6%.^17^ Because GnP frequently causes PN,^6^ the risk of severe PN may increase during mFOLFIRINOX treatment after GnP treatment. Clinicians should be attentive of patients who experience PN during GnP treatment.

The current study had limitations. First, this study was a retrospective study performed at a single referral center. Although the baseline characteristics of the patients were different, mFOLFIRINOX was identified as an independent factor associated with favorable PFS. Another limitation was that the sample size of the study was small. Thus, to clarify the survival benefit of mFOLFIRINOX, further multicenter, large‐scale studies are required.

In conclusion, mFOLFIRINOX as a second‐line regimen contributed to favorable treatment outcomes. Patients treated with mFOLFIRINOX experienced more frequent adverse events than patients treated with S‐1. Additionally, mFOLFIRINOX was identified as an independent factor associated with favorable PFS, suggesting that mFOLFIRINOX could be a promising treatment option for patients with GnP failure.

## References

[1] Siegel RL , Miller KD , Jemal A . Cancer statistics, 2020. CA Cancer J. Clin. 2020; 70: 7–30. 31912902 10.3322/caac.21590

[2] Bray F , Ferlay J , Soerjomataram I , Siegel RL , Torre LA , Jemal A . Global cancer statistics 2018: GLOBOCAN estimates of incidence and mortality worldwide for 36 cancers in 185 countries. CA Cancer J. Clin. 2018; 68: 394–424. 30207593 10.3322/caac.21492

[3] Fukuda J , Ikezawa K , Nakao M *et al*. Predictive factors for pancreatic cancer and its early detection using special pancreatic ultrasonography in high‐risk individuals. Cancers. 2021; 13: 1–15. 10.3390/cancers13030502 PMC7865866 33525645

[4] Yoshioka T , Shigekawa M , Ikezawa K *et al*. Risk factors for pancreatic cancer and the necessity of long‐term surveillance in patients with pancreatic cystic lesions. Pancreas. 2020; 49: 552–60. 32282769 10.1097/MPA.0000000000001521

[5] Khorana AA , Mangu PB , Berlin J *et al*. Potentially curable pancreatic cancer: American Society of Clinical Oncology Clinical Practice Guideline. J. Clin. Oncol. 2016; 34: 2541–56. 27247221 10.1200/JCO.2016.67.5553

[6] Von Hoff DD , Ervin T , Arena FP *et al*. Increased survival in pancreatic cancer with nab‐paclitaxel plus gemcitabine. N. Engl. J. Med. 2013; 369: 1691–703. 24131140 10.1056/NEJMoa1304369 PMC4631139

[7] Conroy T , Desseigne F , Ychou M *et al*. FOLFIRINOX versus gemcitabine for metastatic pancreatic cancer. N. Engl. J. Med. 2011; 364: 1817–25. 21561347 10.1056/NEJMoa1011923

[8] Okusaka T , Ikeda M , Fukutomi A *et al*. Phase II study of FOLFIRINOX for chemotherapy‐naïve Japanese patients with metastatic pancreatic cancer. Cancer Sci. 2014; 105: 1321–6. 25117729 10.1111/cas.12501 PMC4462360

[9] Ozaka M , Ishii H , Sato T *et al*. A phase II study of modified FOLFIRINOX for chemotherapy‐naïve patients with metastatic pancreatic cancer. Cancer Chemother. Pharmacol. 2018; 81: 1017–23. 29633005 10.1007/s00280-018-3577-9

[10] Franck C , Müller C , Rosania R , Croner RS , Pech M , Venerito M . Advanced pancreatic ductal adenocarcinoma: moving forward. Cancers. 2020; 12: 1955. 10.3390/cancers12071955 PMC7409054 32708493

[11] Stein SM , James ES , Deng Y *et al*. Final analysis of a phase II study of modified FOLFIRINOX in locally advanced and metastatic pancreatic cancer. Br. J. Cancer. 2016; 114: 737–43. 27022826 10.1038/bjc.2016.45 PMC4984865

[12] National Comprehensive Cancer Network . NCCN Clinical Practice Guidelines in Oncology: Pancreatic Adenocarcinoma Version 1.2020. Plymouth Meeting, Pennsylvania: National Comprehensive Cancer Network; 2020.

[13] Pelzer U , Schwaner I , Stieler J *et al*. Best supportive care (BSC) versus oxaliplatin, folinic acid and 5‐fluorouracil (OFF) plus BSC in patients for second‐line advanced pancreatic cancer: a phase III‐study from the German CONKO‐study group. Eur. J. Cancer. 2011; 47: 1676–81. 21565490 10.1016/j.ejca.2011.04.011

[14] Wang‐Gillam A , Li C‐P , Bodoky G *et al*. Nanoliposomal irinotecan with fluorouracil and folinic acid in metastatic pancreatic cancer after previous gemcitabine‐based therapy (NAPOLI‐1): a global, randomised, open‐label, phase 3 trial. Lancet. 2016; 387: 545–57. 26615328 10.1016/S0140-6736(15)00986-1

[15] Wang‐Gillam A , Hubner RA , Siveke JT *et al*. NAPOLI‐1 phase 3 study of liposomal irinotecan in metastatic pancreatic cancer: final overall survival analysis and characteristics of long‐term survivors. Eur. J. Cancer. 2019; 108: 78–87. 30654298 10.1016/j.ejca.2018.12.007

[16] Oettle H , Riess H , Stieler JM *et al*. Second‐line oxaliplatin, folinic acid, and fluorouracil versus folinic acid and fluorouracil alone for gemcitabine‐refractory pancreatic cancer: outcomes from the CONKO‐003 Trial. J. Clin. Oncol. 2014; 32: 2423–9. 24982456 10.1200/JCO.2013.53.6995

[17] Sawada M , Kasuga A , Mie T *et al*. Modified FOLFIRINOX as a second‐line therapy following gemcitabine plus nab‐paclitaxel therapy in metastatic pancreatic cancer. BMC Cancer. 2020; 20: 449. 32434547 10.1186/s12885-020-06945-8 PMC7238500

[18] Morizane C , Okusaka T , Furuse J *et al*. A phase II study of S‐1 in gemcitabine‐refractory metastatic pancreatic cancer. Cancer Chemother. Pharmacol. 2009; 63: 313–9. 18398614 10.1007/s00280-008-0741-7

[19] Todaka A , Fukutomi A , Boku N *et al*. S‐1 monotherapy as second‐line treatment for advanced pancreatic cancer after gemcitabine failure. Jpn. J. Clin. Oncol. 2010; 40: 567–72. 20189975 10.1093/jjco/hyq005

[20] Iede K , Yamada T , Kato R *et al*. Efficacy of S‐1 in second‐line chemotherapy after nab‐paclitaxel plus gemcitabine for patients with advanced pancreatic cancer. Cancer Rep. 2020; 3: e1215. 10.1002/cnr2.1215 PMC7941501 32672000

[21] Nakai Y , Isayama H , Sasaki T *et al*. Impact of S‐1 on the survival of patients with advanced pancreatic cancer. Pancreas. 2010; 39: 989–93. 20467352 10.1097/MPA.0b013e3181d91936

[22] Eisenhauer EA , Therasse P , Bogaerts J *et al*. New response evaluation criteria in solid tumours: revised RECIST guideline (version 1.1). Eur. J. Cancer. 2009; 45: 228–47. 19097774 10.1016/j.ejca.2008.10.026

[23] Assaf E , Verlinde‐Carvalho M , Delbaldo C *et al*. 5‐Fluorouracil/leucovorin combined with irinotecan and oxaliplatin (FOLFIRINOX) as second‐line chemotherapy in patients with metastatic pancreatic adenocarcinoma. Oncology. 2011; 80: 301–6. 21778770 10.1159/000329803

[24] Matsumoto T , Kurioka Y , Okazaki U *et al*. FOLFIRINOX for advanced pancreatic cancer patients after Nab‐paclitaxel plus gemcitabine failure. Pancreas. 2020; 49: 574–8. 32282772 10.1097/MPA.0000000000001534

[25] Kanda Y . Investigation of the freely available easy‐to‐use software “EZR” for medical statistics. Bone Marrow Transplant. 2013; 48: 452–8. 23208313 10.1038/bmt.2012.244 PMC3590441

[26] Chung MJ , Kang H , Kim HG *et al*. Multicenter phase II trial of modified FOLFIRINOX in gemcitabine‐refractory pancreatic cancer. World J Gastrointest Oncol. 2018; 10: 505–15. 30595804 10.4251/wjgo.v10.i12.505 PMC6304301

[27] Kobayashi N , Shimamura T , Tokuhisa M , Goto A , Endo I , Ichikawa Y . Effect of FOLFIRINOX as second‐line chemotherapy for metastatic pancreatic cancer after gemcitabine‐based chemotherapy failure. Medicine. 2017; 96: e6769. 28489753 10.1097/MD.0000000000006769 PMC5428587

[28] Kimura G , Takahashi H , Umemoto K *et al*. Efficacy of S‐1 compared to modified FOLFIRINOX as second‐line chemotherapy regimens after gemcitabine plus nab‐paclitaxel for patients with metastatic pancreatic cancer (in abstract). J. Clin. Oncol. 2017; 35: 449–9.

[29] Park W , Chen J , Chou JF *et al*. Genomic methods identify homologous recombination deficiency in pancreas adenocarcinoma and optimize treatment selection. Clin. Cancer Res. 2020; 26: 3239–47. 32444418 10.1158/1078-0432.CCR-20-0418 PMC7380542

[30] Jung JH , Shin DW , Kim J , Lee JC , Hwang JH . Primary granulocyte colony‐stimulating factor prophylaxis in metastatic pancreatic cancer patients treated with FOLFIRINOX as the first‐line treatment. Cancers. 2020; 12: 1–9. 10.3390/cancers12113137 PMC7692712 33120908

[31] Yamao K , Takenaka M , Yoshikawa T *et al*. Clinical safety and efficacy of secondary prophylactic pegylated G‐CSF in advanced pancreatic cancer patients treated with mFOLFIRINOX: a single‐center retrospective study. Intern. Med. 2019; 58: 1993–2002. 30996164 10.2169/internalmedicine.2234-18 PMC6702006

